# Liver cirrhosis with the development of transdiaphragmatic collateral circulation

**DOI:** 10.31744/einstein_journal/2024AI0564

**Published:** 2024-11-21

**Authors:** Lucas Gabriel Annechini Marques, Eduardo Kaiser Ururahy Nunes Fonseca

**Affiliations:** 1 Universidade de São Paulo Department of Radiology São Paulo SP Brazil Department of Radiology, Universidade de São Paulo, São Paulo, SP, Brazil.; 2 Universidade de São Paulo Department of Cardiothoracic Radiology São Paulo SP Brazil Department of Cardiothoracic Radiology, Universidade de São Paulo, São Paulo, SP, Brazil.

A 70-year-old man with liver cirrhosis underwent follow-up examinations at our hospital while awaiting liver transplantation. His medical history included an episode of upper gastrointestinal bleeding secondary to the rupture of esophageal varices approximately a year ago.

**Figure 1:**
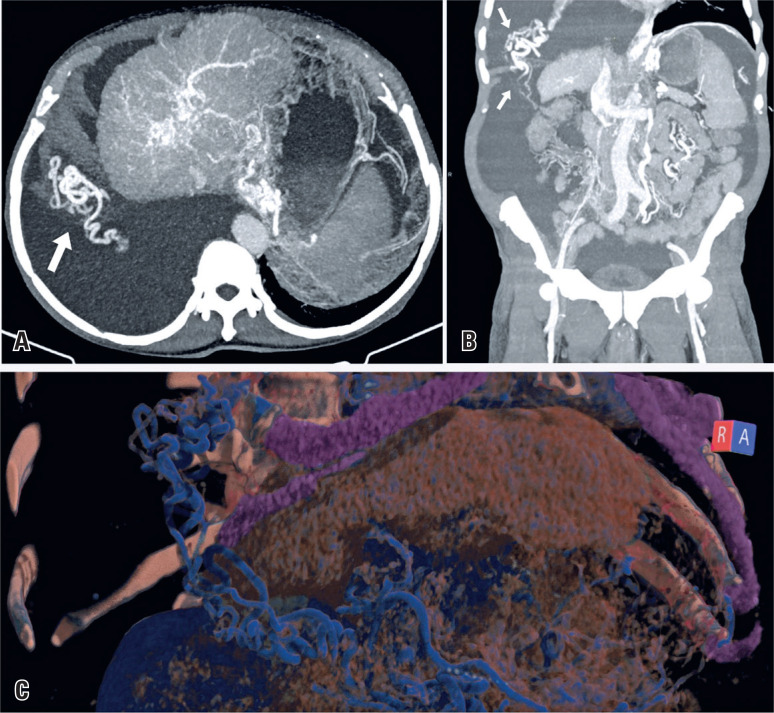
Contrast-enhanced computed tomography reconstruction images in the portal venous phase of a 70-year-old man diagnosed with liver cirrhosis. (A) Axial and (B) coronal maximum intensity projection images reveal signs of chronic liver disease, ascites, and a large pleural effusion in the right hemithorax with vessels piercing through the right hemidiaphragm (white arrows), communicating with the pulmonary circulation. (C) Tridimensional reconstruction showing the collateral vessels ascending through the right hemidiaphragm

His computed tomography (CT) showed signs of chronic liver disease with subacute portal vein thrombosis, cavernous transformation at the abdominal level, ascites, and right pleural effusion. Furthermore, multiple collateral vessels were observed, some of which ascended through the right hemidiaphragm to communicate with the pulmonary veins, which is consistent with transdiaphragmatic collateral circulation (Figure 1).

Transdiaphragmatic collateral circulation is a rare form of shunting in portal hypertension that includes drainage to the pericardiacophrenic, internal thoracic, diaphragmatic, mediastinal, and superior epigastric veins.^(1–3)^

It is related to a loose collateral plexus distributed over the hepatic surface that pierces the diaphragm and communicates with the pulmonary circulation, previously known as the veins of Sappey.^(3,4)^

Its treatment involves reducing the portal system pressure, including transjugular intrahepatic portosystemic shunt and its variations, such as direct intrahepatic portal shunt and intravascular ultrasound.

In liver transplants, portosystemic collaterals should be assessed using serial portal pressure measurements and Doppler ultrasound characterization of the portal flow. To avoid portal steal and graft hypoperfusion, hemodynamically significant collaterals should be ligated during a living donor transplantation. When deceased donor transplantation is considered, collateral ligation should be considered when a combination of low portal flow and large collaterals is observed.^(5)^
